# The Late Mr. Adrian C. F. Hope

**Published:** 1904-05-14

**Authors:** 


### OBITUARY.

### THE LATE MR. ADRIAN C. F. HOPE,

### Secretary of the Hospital for Sick Children, Great Ormond Street.

With the deepest regret we have to announce the death, at St. Thomas's Hospital, on Wednesday morning, of Mr. Adrian Hope, the Secretary of the Hospital for Sick Children, Great Ormond Street. Mr. Hope was admitted to St. Thomas's Hospital on the 2nd inst. and underwent an operation for appendicitis on the 7th inst. It was not generally known that Mr. Hope was unwell, and his death has come as a shock to everybody in the hospital world, where he was well known and highly esteemed.

Mr. Hope became Secretary of the Great Ormond Street Children's Hospital in 1885. Under Mr. Adrian Hope this hospital has been rebuilt, its methods have been modernised and improved, and the financial resources of the hospital have increased by upwards of 100 per cent. In 1888 the total revenue of the hospital was a little over £20,000 a year, and in 1902 the total receipts exceeded £41,000, of which the larger sum was derived from the Imperial Coronation Bazaar, which resulted in a gain of some £19,000 to the hospital exchequer. He believed in endowments, and by strenuous efforts raised the Endowment Fund from £6,000 to £55,000. Mr. Hope's death will therefore be a serious loss to the Great Ormond Street Children's Hospital and all connected with it.

Personally, Mr. Hope was deservedly popular on account of his great charm of manner and originality. He had the wisdom and the heart to take an intelligent interest in the upbringing and training of the younger generation of hospital officials. He was the President of the Hospital Officers' Association, of which he was, we believe, the parent, and had done much to encourage the younger members of the staff of the principal hospitals, and so to materially aid the development of improved methods of hospital administration.

Amongst the secretaries of Metropolitan hospitals Mr. Hope held a high place, and was one of the most influential of hospital secretaries. He married Laura, second daughter of the late Sir Thomas Troubridge, Bart., whose pastel portraits and water colours have brought her much reputation as an artist. We offer Mrs. Hope and her family our sincerest sympathy. Mr. Adrian Hope's death has excited a feeling of personal loss amongst the staff of The Hospital, who had good cause to appreciate his invariable courtesy and the public spirit he displayed in all hospital matters.

